# Effect of Novel Compound LX519290, a Derivative of l-*allo* Threonine, on Antioxidant Potential in Vitro and in Vivo

**DOI:** 10.3390/ijms17091451

**Published:** 2016-09-01

**Authors:** Kun Chun, Md Badrul Alam, Hyeong-U Son, Sang-Han Lee

**Affiliations:** 1 Department of Food Science & Biotechnology, Kyungpook National University, Daegu 41566, Korea; cg6890@hotmail.com (K.C.); mbalam@knu.ac.kr (M.B.A.); vicpie@knu.ac.kr (H.-US.); 2 Research and Development (R&D) Center, Huons Co., Ltd., Ansan 15588, Korea

**Keywords:** l-*allo* threonine, LX519290, antioxidative activity, in vitro, in vivo

## Abstract

We investigated the antioxidative activity of LX519290, a derivative of l-*allo* threonine, in vitro and in vivo. To evaluate the antioxidative activity of LX519290, we performed several in vitro assays (2,2-diphenyl-1-picrylhydrazyl (DPPH) and 2,2′-azino-bis-(3-ethylbenzthiazoline-6-sulphonic acid) (ABTS) radical-scavenging assays, a ferric reducing antioxidant power assay, cupric-reducing antioxidant capacity, and oxygen radical absorbance capacity assay) and evaluated inhibition against the generation of nitric oxide (NO) and reactive oxygen species (ROS) in murine macrophage (RAW264.7) cells. The results showed that LX519290 possessed very strong radical scavenging activity and reducing power, and inhibited NO and ROS generation in a dose-dependent manner without showing any cytotoxicity. LX519290 treatment also increased the total thiol content and glutathione *S*-transferases (GST) activities in RAW264.7 cells. Finally, we also determined whether LX519290 affects the mRNA levels of antioxidant enzymes in vitro and in vivo. The expression of superoxide dismutase (SOD), glutathione peroxidase (GPx), and catalase (CAT) were markedly higher in the sample-treated group than in the oxidative stress group. LX519290 treatment also increased the transcriptional and translational activities of NF-E2-related factor-2 (Nrf-2) with corresponding increases in the transcriptional and translational activities of haeme oxygenase-1 (HO-1). Collectively, the data demonstrated that LX519290 has potent antioxidative activity, decreases NO and ROS generation, increases total thiol content and GST activities in RAW264.7 cells, and increases the transcriptional and translational levels of antioxidant enzymes in vitro and in vivo.

## 1. Introduction

Toxic reactive oxygen species (ROS) are generated during aerobic metabolism, and excessive generation of ROS, which include superoxide anions (O_2_^•−^), hydrogen peroxide (H_2_O_2_), and hydroxyl radicals (OH^•^), causes oxidative stress [1]. Oxidative stress is mainly associated with the pathogenesis of various disorders and diseases, such as diabetes, cancer, and cardiovascular disorders, and accordingly, interest has recently been increasing in the use of antioxidants for the maintenance of human health and prevention and treatment of certain diseases [2]. Antioxidants have shown a role in cellular protection against oxidative stress through direct or indirect pathways depending upon their working mechanism. In direct pathways, antioxidants scavenge reactive oxygen and nitrogen species by being consumed or chemically modified. In contrast, indirect pathways are involved by upregulating phase II detoxifying and antioxidative enzymes [3].

**Figure 1:**
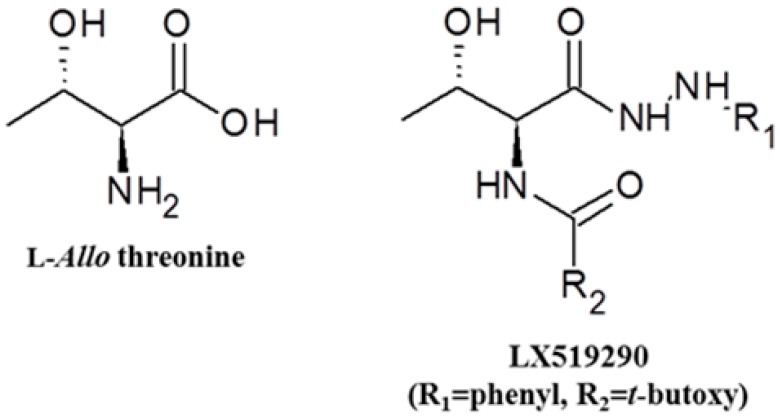
Chemical structure of LX519290. LX519290 was synthesized using the following procedure: A solution of l-*allo* threonine (1.0 equivalent volume) in methyl alcohol was added to acetyl chloride (3.0 equivalent volume) dropwise at 0 °C. The mixture was permitted to warm gently to reflux, evaporate, crystallize to produce l-*allo* threonine methyl ester as a white powder. The subsequent product (1.0 equivalent volume) and sodium hydroxide (2.5 equivalent volume) in dioxane/water (1:1, *v*/*v*) was cooled to 0 °C and treated portionwise with di-*tert*-butyldicarbonate (1.1 equivalent volume) over 10 min, then stirred at 0 °C for 2 h, warmed to 25 °C, and concentrated. The product was next partitioned between ethyl acetate and water, thereafter, the organic layer was collected, dried over MgSO_4_, and concentrated. The residual fraction was purified by chromatography to allow a white solid carbamic acid *tert*-butyl ester. A solution of the resulting product (1.0 equivalent volume) and phenyl hydrazine (10.0 equivalent volume) in ethanol was refluxed at 100 °C for 1 h. The solution was stirred at 25 °C, and then the concentrated crude product was partitioned, and saturated with sodium hydroxide for collecting organic layer. The residue was purified by chromatography to afford a white powder LX519290 (R_1_ = phenyl, R_2_ = *tert*-butoxy). After these steps using l-*allo* threonine, LX519290 was attained. LX519290 has a unique structure that consists of C_15_H_23_N_3_O_4_ with a molecular weight of 309.325.

Aerobic organisms have effective antioxidant networks to defend against oxidative stress, involving primary enzymes such as superoxide dismutase (SOD), catalase (CAT), and glutathione peroxidase (GPx), as well as inducible phase II detoxifying enzymes such as haeme oxygenase-1 (HO-1) and NAD(P)H:quinone oxidoreductase1 (NQO1) through the activation of NF-E2-related factor-2 (Nrf-2) [4]. Moreover, some antioxidants have displayed their antioxidant activity in both a direct and indirect fashion and are referred to as bifunctional antioxidants [5]. For this reason, we studied the antioxidative effects of LX519290, a derivative of l-*allo* threonine (Figure 1), using in vitro and in vivo assays, to evaluate whether it has potency as a bifunctional antioxidant to lessen oxidative stress.

LX519290 has been previously screened by Heo et al. for anti-atopic and anti-asthmatic activity [6,7]. The amino acid, l-*allo* threonine, another diastereoisomer of l-threonine, is not naturally existing in the human whole body and is a component of globomycin which is a peptide antibiotic exhibiting spheroplast-forming activity [8]. It is known that the amino acid can be biosynthesized by acetaldehyde and glycine in a serine hydroxymethyl transferase-catalysed aldol reaction in *Escherichia coli* [9]. Although the metabolic pathway of l-*allo* threonine in the human being remains unidentified, research has shown that it cannot be digested into l-threonine in chickens [10].

In the current study, we investigated the antioxidative activity of LX519290 in vitro and in a 2,2′-azobis(2-methylpropionamidine) dihydrochloride (AAPH)-induced oxidative stress animal model.

## 2. Results

### 2.1. Radical-Scavenging Effects of LX519290 in Vitro

**Figure 2:**
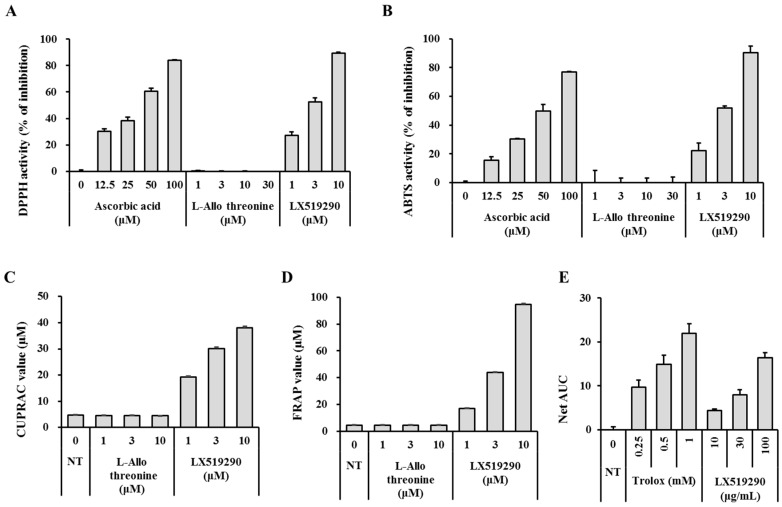
Radical scavenging effects of LX519290 in vitro. The 2,2-diphenyl-1-picrylhydrazyl (DPPH) radical scavenging assay (**A**); 2,2′-azino-bis-(3-ethylbenzothiazoline-6-sulphonic acid) (ABTS^•+^) radical scavenging assay (**B**); ferric reducing antioxidant power (FRAP) assay (**C**); and cupric-reducing antioxidant capacity (CUPRAC) assay (**D**) were conducted with various concentrations of LX519290, and ascorbic acid was tested as a standard antioxidant compound; (**E**) The oxygen radical absorbance capacity (ORAC) activities of the samples were measured by subtracting the area under the blank curve from the area under the sample curve, to gain the net area under the curve (Net fluorescence decay curve (AUC)). NT: not treated.

To assess in vitro antioxidant activity, we first examined whether LX519290 possessed free radical scavenging activity by using DPPH^•^ and ABTS^•+^ scavenging assays. LX519290 reduced the DPPH^•^, a stable organic nitrogen radical, in a dose dependent fashion (Figure 2A), and it was shown that LX519290 has a higher antioxidant activity than its precursor l-*allo* threonine. Furthermore, in the decrease of the radical cation from 2,2′-azino-bis-(3-ethylbenzothiazoline-6-sulphonic acid) (ABTS^•+^), a combined electron transfer and hydrogen atom transfer assay, LX519290 also showed higher antioxidant activity than l-*allo* threonine (Figure 2B).

Next, we used ferric reducing antioxidant power (FRAP) and cupric-reducing antioxidant capacity (CUPRAC) assays to assess whether LX519290 has an electron donating capacity. The FRAP test is a pure electron transfer assay in which ferric ions are reduced by antioxidants that are detected by the formation of a complex with the probe di-2,4,6-tripyridyl-s-triazine (TPTZ) [11]. Moreover, in the CUPRAC assay, the chromogenic oxidizing agent bis(neocuproine)copper(II) chloride (Cu(II)-Nc) reacts with *n*-electron reductant antioxidants and is reduced to the highly colored Cu(I)-Nc chelate showing maximum absorption at 405 nm [12]. In this study, LX519290 showed an appreciably higher reduction capacity than l-*allo* threonine (Figure 2C,D).

The oxygen radical absorbance capacity (ORAC) assay utilizes an AAPH-induced peroxyl radical that mimics lipid peroxyl radicals comprised of the lipid peroxidation chain reaction in vivo. Inhibition of peroxyl radical-induced oxidation of a fluorescent probe, fluorescein, by antioxidants was continually monitored and the protective effect of an antioxidant was measured by evaluating the area under the fluorescence decay curve (AUC) [13]. Net AUC values of Trolox and LX519290 were dose-dependently increased (Figure 2E, columns 5–7) and these results indicated that LX519290 (30 µM) exerted similar antioxidant activity to 0.5 mM Trolox (Figure 2E, comparing columns 2–4 with columns 5–7).

### 2.2. Cell Viability of LX519290

**Figure 3:**
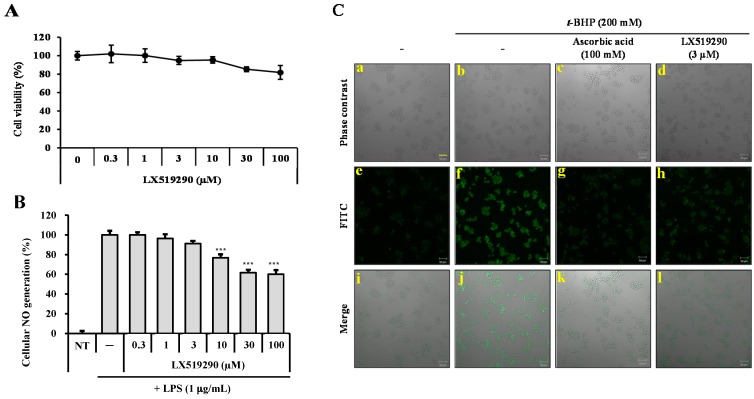
Cell viability and inhibition of nitric oxide (NO) and reactive oxygen species (ROS) generation in RAW264.7 cells. RAW264.7 cells were split and seeded at a density of 2 × 10^4^ cells per well (96-well plate) and a 3-(4,5-dimethylthiazol-2-yl)-2,5-diphenyltetrazolium bromide (MTT) assay (**A**); Griess reagent (**B**); and 2′,7′-dichlorofluorescin diacetate (DCFH-DA) assay (**C**) were conducted with various concentrations of LX519290. *** *p* < 0.001 indicates a significant difference from the negative control, using a one-way ANOVA followed by Tukey’s post hoc test. RAW264.7 cells were cultured and treated with (**b**–**d**,**f**–**h**,**j**–**l**) or without (**a**,**e**,**i**) *tert*-butyl hydroperoxide (*t*-BHP) and/or ascorbic acid (**c**,**g**,**k**)/LX519290 (**d**,**h**,**l**), as described in the Materials and Methods section. Phase contrast (**a**–**d**), FITC (**e**–**h**), and merged (**i**–**l**) images were photographed to measure ROS generation. Scale bar: 50 μm.

The effects of LX519290 on cell viability in RAW264.7 cells were comprehensively investigated (Figure 3A). Cell viability with various doses (0.3–100 µM) of LX519290 was measured with a 3-(4,5-dimethylthiazol-2-yl)-2,5-diphenyltetrazolium bromide (MTT) assay kit. The mitochondrial enzyme activity was taken as an indirect measure of the amount of viable cells. The results showed no cytotoxic effects at doses up to 100 µM.

### 2.3. Inhibition Effects of LX519290 on Cellular Nitric Oxide (NO) and Reactive Oxygen Species (ROS) Generation in RAW264.7 Cells

In cells stimulated with lipopolysaccharide (LPS) or other stimuli, the intracellular ROS level in macrophages increased rapidly, causing oxidative stress. We then tried to evaluate the antioxidant activity of LX519290 by examining NO generation and ROS generation. The LPS stimulation significantly increased the accumulation of NO to 121.9% in the medium compared with untreated control cells, whereas pretreatment with LX519290 notably decreased NO release by 23.2% (*p* < 0.001), 38.4% (*p* < 0.001), and 40.0% (*p* < 0.001) at doses of 10, 30, and 100 µM, respectively (Figure 3B). To further examine the effects of LX519290 on the ROS generation, *tert*-butyl hydroperoxide (*t*-BHP)-induced RAW cells were analyzed by monitoring cell morphology. The results showed that LX519290 pretreatment notably decreased *t*-BHP-induced ROS generation (Figure 3C). Ascorbic acid (a positive control) also exhibited potential in inhibiting cellular ROS generation (Figure 3C, comparison of f–h and of j–l). These results indicate that LX519290 inhibited intracellular ROS as well as NO generation without any cellular toxicity.

### 2.4. Effects of LX519290 Against Pro-Oxidant Challenge in RAW264.7 Cells

**Figure 4:**
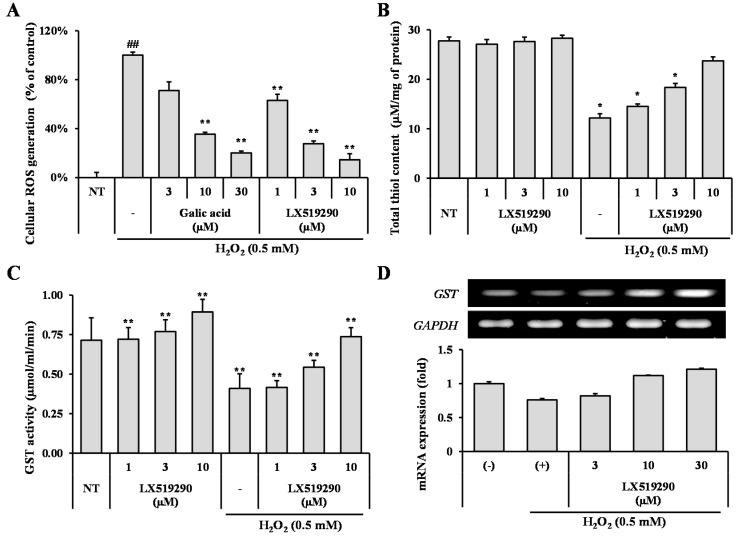
Effect of LX519290 against pro-oxidant challenge in RAW264.7 cells. RAW264.7 cells were split and seeded at a density of 5 × 10^4^ cells per well (96-well plate) and measured ROS generation (**A**); total thiol content (**B**); glutathione *S*-transferases (GST) activity (**C**); and its mRNA expression (**D**) which were conducted with various concentrations of LX519290. ^##^ *p* < 0.01 indicates a significant difference from the no treatment group; * *p* < 0.05 and ** *p* < 0.01 indicate a significant difference from the H_2_O_2_-treated group.

To illustrate, whether LX519290 has activity against pro-oxidant challenge, an H_2_O_2_ induced cellular ROS generation assay was carried out. The increases in the ROS level seen are indicative of intracellular oxidative stress in cells treated with H_2_O_2_ compared with untreated cells (Figure 4A); however, LX519290-treatment, as expected, ameliorated ROS generation by up to 37% (*p* < 0.01), 72% (*p* < 0.01), and 85% (*p* < 0.01) at the 1, 3, and 10 µM doses, respectively, as compared with H_2_O_2_-treated cells (Figure 4A).

To further scrutinize the possible role of LX519290 in protein thiol regulation, we next focused on cellular thiol content measurement under pro-oxidant challenge conditions. In non-treated cells, LX519290 did not show any significant change in cellular thiol content. In contrast, after pro-oxidant challenge, the cellular thiol content was drastically decreased as compared to that of the non-treated cell, whereas LX519290 treatment increased the total thiol content in the cells concentration-dependently (Figure 4B). Furthermore, we also demonstrated the effects of LX519290 on the glutathione *S*-transferases (GST) activity of cells and found that LX519290 has the potential to increase GST activity in both normal as well as oxidative stress conditions (Figure 4C,D).

### 2.5. Effects of LX519290 on Gene Expressions of Antioxidants and Phase II Antioxidant Enzymes

**Figure 5:**
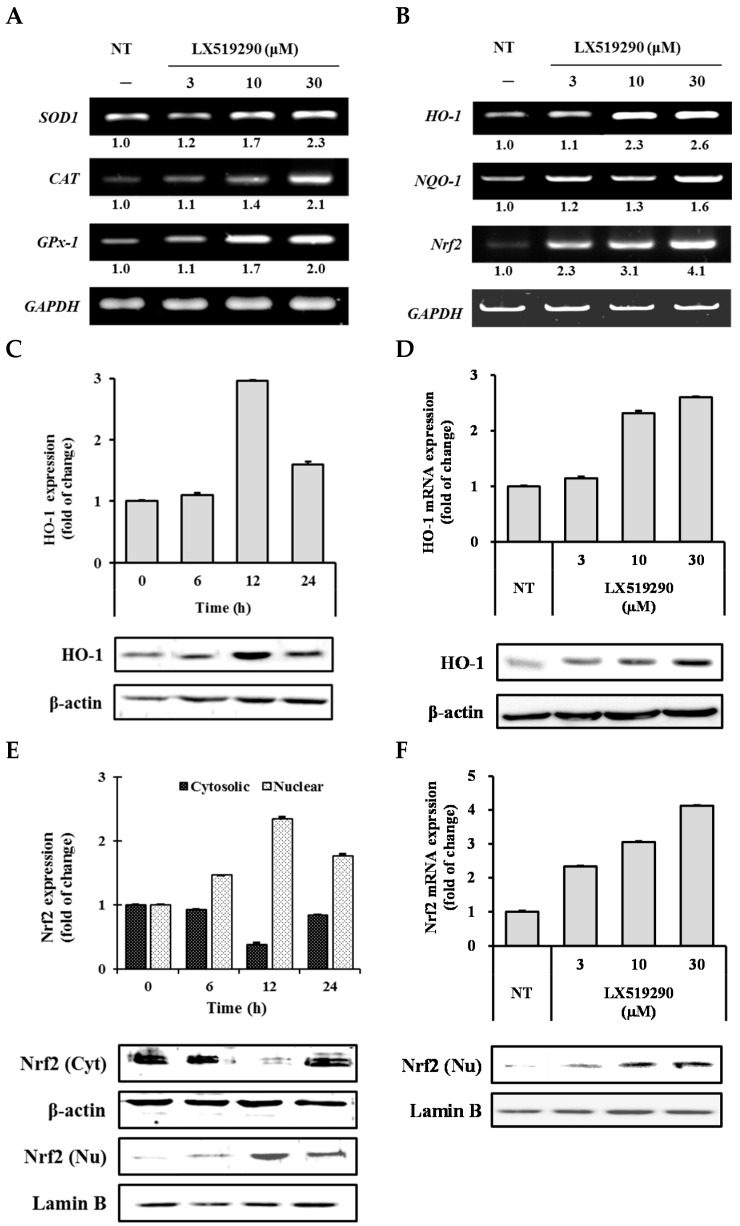
Analysis of primary and Phase II antioxidant and detoxifying enzyme mRNA levels in RAW264.7 cells. RAW264.7 cells were pretreated for 24 h with specified concentrations of LX519290. The mRNA expression of primary antioxidant enzyme (**A**); and phase II antioxidant and detoxifying enzyme (**B**) were measured by reverse transcription polymerase chain reaction (RT-PCR). The protein level of haeme oxygenase-1 (HO-1) in time dependent (**C**); and concentration dependent (**D**); and the nuclear translocation of NF-E2-related factor-2 (Nrf2) in time dependent (**E**); and concentration dependent (**F**) were analyzed by Western blot.

To investigate the effects of LX519290 on antioxidant enzymes (SOD1, CAT, and GPx-1) and phase II detoxifying enzymes (HO-1 and NQO1), RAW264.7 cells were treated with LX519290 for 24 h and the transcription levels of *SOD1*, *CAT*, *GPx-1*, *HO-1*, and *NQO1* genes were examined. PCR analysis indicated a dose dependent increase in the mRNA expression of *SOD1*, *CAT*, and *GPx-1*, (Figure 5A) as well as *HO-1* and *NQO1* (Figure 5B). The protein level of HO-1 was further confirmed by Western blot analysis (Figure 5C,D).

Nrf2 has been reported to be a crucial nuclear factor stimulating phase II enzymes. To confirm that LX519290 activates these phase II enzymes through Nrf2, the transcription level of the *Nrf2* gene was analyzed and it was found that LX519290 significantly increased *Nrf2* mRNA expression (Figure 5B). The protein levels of Nrf2 in the nucleus and the cytosol were also examined, and a time course study revealed that treatment with LX519290 caused time dependent increases of Nrf2 translation into the nucleus with a peak level at 12 h after treatment (Figure 5E).

### 2.6. In Vivo Antioxidant Activity of LX519290

**Figure 6:**
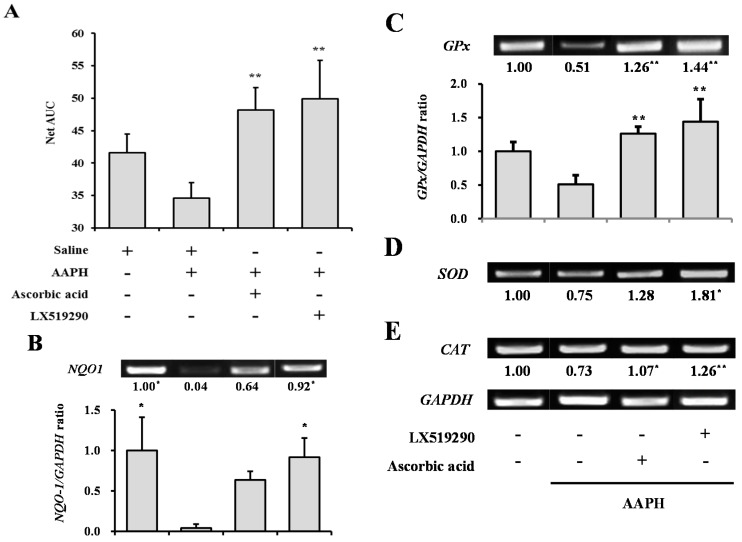
The ORAC activity in the plasma after intraperitoneal injection of LX519290. (**A**) Rats were randomly divided into four groups (*n* = 5 rats per group): a normal group (saline), negative control group (AAPH, 50 mg/kg body weight), positive control group (ascorbic acid, 150 mg/kg body weight), and LX519290 group (15 mg/kg body weight). ** *p* < 0.01, significantly different from AAPH-treated group, using a one-way ANOVA followed by Tukey’s post hoc test. Analysis of mRNA levels of NAD(P)H:quinone oxidoreductase1 (*NQO1*), glutathione peroxidase (*GPx*), superoxide dismutase (*SOD*), and catalase (*CAT*) in liver tissue. Male Wistar rats, aged 7–8 weeks and weighing 230–270 g (Samtaco Korea, Osan, Korea), were administered intraperitoneal AAPH with LX519290 for 10 days. The liver was excised from each animal, and the mRNA level of *NQO1* (**B**); *GPx* (**C**); *SOD* (**D**); and *CAT* (**E**) was measured by RT-PCR. * *p* < 0.05, ** *p* < 0.01 indicates a significant difference from the AAPH-treated group, using a one-way ANOVA followed by Tukey’s post hoc test.

Figure 6 shows ORAC activity in the plasma after intraperitoneal administration of AAPH and antioxidant compounds for 10 days. The blood samples from the LX519290-treated group showed the highest Net AUC values. The AAPH-treated negative control group showed a lower Net AUC value (16.7%) than the saline-treated NT group. The ascorbic acid- or LX519290-treated groups showed increased Net AUC values, by 1.39-fold and 1.44-fold (*p* < 0.01) respectively, from the value for the negative control group (Figure 6A).

We further examined whether mRNA expression levels of *SOD1*, *CAT*, *GPx*, and *NQO1* were altered in rat liver tissue by treatment with LX519290 for 10 days. As shown in Figure 6, treatment with 50 mg/kg AAPH decreased mRNA expression levels of the antioxidant enzymes, whereas administration of LX519290 significantly changed the mRNA levels of *SOD1* (141.9%; *p* < 0.05), *GPx* (182.6%; *p* < 0.01), and *CAT* (72.1%; *p* < 0.01), which were markedly higher than those of the AAPH-treated group, and *NQO1* (91.6%; *p* < 0.01) mRNA recovered to the normal level.

## 3. Discussion

ROS, including superoxide anion (O_2_^•−^), hydrogen peroxide (H_2_O_2_), and hydroxyl radical (OH^•^), are by-products of cellular metabolic reactions and pathways, and are intracellular, toxic species, partially produced by reduction of oxygen (O_2_). It is well documented that ROS are more chemically reactive than O_2_; thus, ROS were thought to act entirely as cellular harmful agents, extensively reacting with lipids, proteins, and DNA [14]. Previously, we produced a unique chemical library using a combinatorial chemistry technique. LX519290 was a hit derived from this library and exhibited anti-atopic dermatitis [6] and anti-asthmatic activity [7]. Therefore, this protective effect may confer an avenue for preventive and curative approaches by anti-oxidation and anti-inflammation. In this regard, we examined the antioxidant effects of LX519290 using various in vitro and in vivo methods to hypothesize a possible mechanism.

The compounds become an antioxidant if they (i) have a hydrogen or electron-donating capacity, (ii) have the ability to stabilize and delocalize the unpaired unstable electron, and (iii) have metal-chelating potential [15]. DPPH^•^ and ABTS^•+^ are considered the most popular spectrophotometric methods for the determination of the antioxidant capacity of any test molecule. In the DPPH assay, the stable free radical DPPH accepts an electron or hydrogen radical from antioxidant and reduces to become a stable diamagnetic non-radical DPPH-H molecule [16] with consequent discolouration and decrease in absorbance. The degree of discolouration directly correlates with the magnitude of scavenging potential of an antioxidant compound in terms of its hydrogen donating ability. Moreover, in the ABTS^•+^ scavenging assay, the pre-formed ABTS^•+^ is generated by oxidation of ABTS with potassium persulphate, which can be reduced in the presence of hydrogen-donating antioxidants. As presented in Figure 2, LX519290 exhibited more effective radical cation scavenging activity than its precursor, in agreement with previous results [11,17], indicating that the presence of phenylhydrazine as well as the character of the side chain made a significant difference, possibly owing to the ability to donate electrons or hydrogen atoms. FRAP assay involves the reduction of yellow ferric tripyridyltriazine complex (Fe(III)-TPTZ) to blue ferrous complex(Fe(II)-TPTZ) by the action of electron donating antioxidant which are measured spectrophotometrically at 593 nm [18]. The ability of a compound to generate Fe(II) from Fe(III) defined as “antioxidant power” in the FRAP assay because some antioxidants, such as ascorbic acid, can decrease both reactive species and Fe(III) and their ability in lessening Fe(III) may reflect their ability in diminishing reactive species. Again, the CUPRAC assay uses the copper(II)-neocuproine agent as the chromogenic oxidant. Because of the lower redox potential of the CUPRAC reagent, reducing sugars and citric acid are not oxidized with the CUPRAC reagent [12], therefore, copper reduction may be an even more sensitive indicator to measure antioxidant potentiality [13]. In this study, the results demonstrated noticeable antioxidant potential (in terms of FRAP and CUPRAC, measured as Ascorbic acid equivalent) of LX519290, which was gradually increased with increasing concentrations of samples (Figure 2C,D). The ORAC method in particular is considered a favorable method, owing to its biological relevance to in vivo antioxidant efficacy [19]. In the improved ORAC assay, AAPH loses a dinitrogen to create an AAPH radical that further reacts with oxygen quickly to make a more stable peroxyl radical, ROO^•^. In the presence of antioxidants, ROO^•^ accepts a hydrogen atom from the antioxidant to form ROOH and a steady antioxidant radical. As a result, the injury to fluorescein, caused by the peroxyl radical, is inhibited, and the reaction mechanism was decided to follow the hydrogen atom transfer mechanism [20]. In this study, the net AUC values of LX519290 were increased in a dose-dependent manner, similar to Trolox (Figure 2E), suggesting that LX519290 exhibited strong antioxidant activities in the in vitro assay methods and may possibly follow the hydrogen atom transfer mechanism. NO is a reactive nitrogen species (RNS). The interaction of NO with ROS causes the production of several RNS, including nitric oxide, nitrogen dioxide, and peroxynitrite, that potentiate cellular damage [21]. Therefore, the decrease in NO generation after LX519290 treatment indicates its antioxidant potential.

*t*-BHP is normally used as a model substance for evaluation of mechanisms of cellular modifications resulting from oxidative stress in cells and tissues. *t*-BHP induced oxidative stress not only through the production of peroxyl and alkoxyl radicals but also the depletion of GSH and the initiation of lipoperoxidation of membrane phospholipids with subsequent alterations to membrane fluidity and permeability [22]. However, hydrogen peroxide (H_2_O_2_) is generated closely from all sources of oxidative stress and can diffuse easily in and out of various kinds of cells and tissues. It is also an important cause of oxidative injury due to its longer half-life than other ROS and can simply transform into a hydroxyl radical, one of the most harmful free radicals [23]. 2′,7′-Dichlorofluorescin diacetate (DCFH-DA) diffused into the cells where cellular esterases cleaved the diacetate moiety to make more polar DCFH, which was trapped within the cells. Under oxidative stress conditions, diverse ROS such as superoxide, hydroxyl radicals, hypochlorous acid (HOCl) are closely involved to oxidize the intracellular DCFH to the fluorescent DCF and the level of fluorescence determined upon excitation is related to the level of oxidation. Antioxidants mitigate the production of total ROS by donating either hydrogen or electron to the oxidants, and prevent the oxidation process of DCFH-DA to DCF and, finally, competitively inhibit the increase of fluorescence signal [24,25]. In this study, treatment with LX519290 strongly inhibited the production of cellular ROS production (Figure 3C, comparison of f–h and Figure 4A). The thiol functional group plays an important role in intracellular antioxidant defense systems by directly reacting with some ROS and RNS; therefore, solvent-exposed thiols within cells may contribute to endogenous antioxidant defense systems [26]. High thiol levels may protect cellular proteins against oxidation either via the thiol redox cycle or by directly detoxifying the ROS generated by exposure to stressor agents [27]. Here, LX519290 treatment increased the cellular thiol content in a dose dependent manner (Figure 4B). GST belongs to a group of detoxification enzymes that also require intracellular thiol tripeptide GSH for their catalytic activity [28], and LX519290 treatment significantly increased the cellular GST activity (Figure 5C) as well as its transcription level (Figure 4D), which conclusively demonstrates the antioxidant properties of LX519290.

ROS scavenging activity plays a decisive role in cellular homeostasis during cell proliferation and maintenance. Several enzymes SOD (EC 1.15.1.1), GPx (EC 1.11.1.9), and CAT (EC 1.11.1.6) are associated with the removal of these free radical species within cells. If these enzymes are damaged by several occurrences of oxidative stress, degenerative diseases can result [29]. Cytosolic superoxide (O_2_^−^) is produced by the one-electron reduction of O_2_ through the slippage of electrons from the electron carriers of the mitochondrial electron transport chain. It is well known that O_2_^−^ is rapidly converted into H_2_O_2_ by SOD. Additionally, H_2_O_2_ can be detoxified to H_2_O by the scavenging enzymes, GPx and CAT. These enzymes act together in the metabolic pathway of free radicals [14,30]. In this study, LX519290 treatment significantly increased the mRNA level of antioxidant enzymes such as *SOD1*, *CAT*, and *GPx-1* (Figure 6A) in RAW264.7 cells, revealing that LX519290 has the ability to maintain cellular homeostasis and to protect the cell from oxidative stress.

Under normal condition, Nrf2 is generally tethered in the cytoplasm by the Keap1 protein, and plays a pivotal role in the activation of phase II enzymes, which can be achieved when Nrf2 is set free and translocated to the nucleus by electrophiles and antioxidants [31]. We showed that LX519290 treatment was able to increase the mRNA level of *Nrf-2* (Figure 5B), as well as its translocation into the nucleus (Figure 4E,F), which increased the transcriptional and translational level of phase II antioxidant and detoxifying enzyme HO-1, (Figure 5B–D, respectively) in RAW264.7 cells, in agreement with previous results [32]. This indicated that the antioxidant activity of LX519290 in RAW 264.7 cells is in part attributable to induction of HO-1, which is regulated by the activation of its transcriptional factor Nrf2. HO-1, encoded by the *HMOX1* gene, can alter haeme into the strong pro-oxidant biliverdin, which is then transformed into bilirubin, a potent antioxidant [31]. We hypothesize that the mechanism of interaction between LX519290 and Nrf2 may mimic the action of additional Nrf2 inducers such as sulphoraphane and 5-*O*-caffeoylquinic acid, controlling Nrf2 nuclear translocation and antioxidative responsive element (ARE)-dependent gene expression such as that of *HO-1*, *Nrf2*, and *NQO-1* in HT29 cells [33] and SH-SY5Y cells [34].

To establish the oxidative stress model, we used a broadly reported generator of free radicals, AAPH. AAPH is a water-soluble azo small molecule and decomposition of AAPH produces 1 mole of nitrogen and 2 moles of carbon radicals. The carbon radicals could either combine to generate stable products or react with molecular oxygen to produce peroxyl radicals [35]. As shown in Figure 6A, the blood plasma net AUC value of the LX519290-treated group was dramatically higher than that of the AAPH-treated negative control group. Additionally, the liver REDOX system was characterized by measurement of the activity of hepatic antioxidant enzymes such as CAT, GPx, and SOD [36]. As shown in Figure 6, it was evident that LX519290 increased mRNA expression levels of *SOD1*, *GPx*, and *CAT* from the levels seen in the AAPH-treated group. Furthermore, NQO1 catalyzes two-electron reduction and detoxification of quinones and/or their derivatives, and represents a cytoprotective mechanism against oxidative damage. NQO1 is well known to sustain both α-tocopherol and coenzyme Q_10_ in their decreased antioxidant state [37]. LX519290 significantly changed the mRNA levels of hepatic *NQO1*, which recovered to the normal level in mice after the administration of AAPH (Figure 6). The data showed that the compound exerted potent activity to ameliorate oxidative damage by scavenging free radicals. As a result, we assumed that LX519290 increased the ORAC values for blood plasma, which was likely due to the increases in the erythrocyte antioxidant enzymes: SOD and GPx [38]. The main defense mechanisms for the prevention of liver damage enable the reduction of ROS by increasing antioxidant enzyme activity [39]. Many studies have shown that the excessive ROS production induced by CCl_4_ breaks the balance between ROS production and antioxidant defenses [40,41,42]. Recently, induction of hepatic SOD, CAT, and GPx activity by ursolic acid has been demonstrated in a CCl_4_-treated mouse model [43].

In conclusion, the present study clearly demonstrates that LX519290 has a stronger antioxidant activity than its parental l-allo-threonine in both electron-transfer and hydrogen atom transfer assays, and has the potential to prevent H_2_O_2_-induced oxidative stress damage in RAW264.7 cells through increases in cellular thiol content, and GST activity, as well as the expression of antioxidant enzymes, the redox-sensitive transcription factor *Nrf-2* inducible phase II antioxidant, and detoxifying enzymes such as *HO-1* and *NQO-1*. Moreover, LX519290 also has the potential in safeguarding against AAPH-induced oxidative stress damage in an in vivo animal model, as confirmed by increasing ORAC values and mRNA expression of antioxidant enzyme related-genes, including *SOD*, *GPx*, *CAT*, and *NQO1*. Therefore, the identification of novel cytoprotective agents such as LX519290 against oxidant damage, through their prospective implications, may help to better understand its activity against the several pathophysiological conditions associated with oxidative stress.

## 4. Materials and Methods

### 4.1. Materials

All chemicals, solvents, reagents, and standards used in the experiments were purchased from Sigma Chemical Co. (St. Louis, MO, USA). All solutions were freshly prepared with distilled water. RAW264.7 murine macrophage cell line was purchased from American Type Culture Collection (ATCC; no. TIB-71, Manassas, VA, USA). The detailed protocol used for preparing the compound LX519290 is described elsewhere [44,45].

### 4.2. Animals and Care

Male Wistar rats, aged 7–8 weeks and weighing 230–270 g (Samtaco Korea, Osan, Korea), were used in the experiments. Animals were maintained in an air-conditioned room at a temperature of 22 ± 1 °C and a humidity of 55% ± 1% with a 12 h light/dark cycle. They were fed a standard commercial rodent pellet diet (Samtaco Korea), and had ad libitum access to water. The animal tests complied with Guiding Principles for the Care and Use of Animals and the Guidelines of the Committee of the International Association for the Study of Pain Research and Ethical Issues [46]. All animals were acclimatized to the laboratory environment for at least 1 week prior to testing under the Guidelines of the Committee on Laboratory Animal Ethics, Kyungpook National University (Approved IRB #2013-0095, Daegu, Korea; Date: 11 December 2013). Animals were randomly divided into 4 groups each containing 5 mice. All procedures performed in studies involving animals were in accordance with the ethical standards of the institution or practice at which the studies were conducted. This article does not contain any studies with human participants performed by any of the authors. Informed consent was obtained from all individual participants included in the study.

### 4.3. Radical-Scavenging Activity Assays

The 2,2-diphenyl-1-picrylhydrazyl (DPPH) radical-scavenging assay was used for evaluation of the free radical scavenging activity of LX519290 and was conducted following a previously described protocol [47], with a minor modification. Briefly, 198 μL of a 0.2 mM solution of DPPH in 50% ethanol was added to 2 μL of various concentrations of the sample. The mixture was allowed to stand at 25 °C for 10 min and the absorbance was measured at 517 nm in a microplate reader (Victor3, PerkinElmer, Turku, Finland). Ascorbic acid was checked as a standard compound. The ability to scavenge the DPPH radical was estimated using the following equation:

(1) $$\mathrm{DPPH}\;\text{radical-scavenging}\;\mathrm{activity}\;\left(\%\right)=[\frac{\left({Abs}_{\text{control}}-{Abs}_{\text{sample}}\right)}{{Abs}_{\text{control}}}]\times 100$$

where *Abs*_control_ is the absorbance of the control and *Abs*_sample_ is the absorbance of the sample. All samples were analyzed in triplicate.

The method by Re et al. [48] was adopted for the ABTS assay with slight modifications. Various concentrations of the sample were allowed to react with 198 μL of the ABTS^•+^ solution, and the absorbance was measured at 734 nm. Ascorbic acid was tested as a positive antioxidant compound. The ability to scavenge the ABTS^•+^ was calculated using the following equation:

(2) $${\text{ABTS}}^{•+}\text{-scavenging activity}\;\left(\%\right)=[\frac{\left({Abs}_{\text{control}}-{Abs}_{\text{sample}}\right)}{{Abs}_{\text{control}}}]\times 100$$

where *Abs*_control_ is the absorbance of the control and *Abs*_sample_ is the absorbance of the sample. All samples were analyzed in triplicate.

For the measurement of reducing power, the ferric reducing antioxidant power (FRAP) assay was carried out, as described previously [49]. Two microliters of the aqueous sample at varying concentrations and 198 μL of FRAP reagent were mixed, and the absorbance was recorded at a 595 nm. Ascorbic acid was also used as a standard compound.

The cupric-reducing antioxidant capacity (CUPRAC) of LX519290 was determined according to a previously described assay [12]. A solution of 10 mM CuCl_2_, 7.5 mM neocuproine, and 1 M ammonium acetate buffer (pH 7.0) was added and the resultant solution was mixed to the samples. Following a 1-h incubation period at 25 °C, the absorbance was measured at 450 nm. Ascorbic acid was tested as a standard compound.

### 4.4. Oxygen Radical Absorbance Capacity Assay

The oxygen radical absorbance capacity (ORAC) assay was carried out according to a previous report [13]. Trolox (a water-soluble analog of Vitamin E) was used as a positive control. The experiment was conducted at 37 °C under pH 7.4 conditions with a blank sample in parallel. The analyzer was set to record the fluorescence of 200 nM fluorescein every minute after the addition of 20 mM AAPH with a 480 nm excitation and a 520 nm emission wavelength. The results were calculated using the differences in the areas under the fluorescence decay curves between the blank sample and experimental sample, and were expressed as area under the curve (Net AUC) values.

### 4.5. Cell Viability

Cell viability was assessed using the 3-(4,5-dimethylthiazol-2-yl)-2,5-diphenyltetrazolium bromide (MTT) assay as described previously [50]. For the assay, RAW264.7 cells were split and seeded into a 96-well flat bottom microplate at a density of 2 × 10^4^ cells per well and incubated at 37 °C for 1 h. The cells were then treated with different concentrations of LX519290. After 24 h of incubation, 20 μL of MTT (5 mg/mL in phosphate-buffered saline (PBS) solution was added to each well, and the plate was incubated for another 2 h. Absorbance values were then measured at 450 nm using a microplate reader (Victor3, PerkinElmer).

### 4.6. Measurement of Cellular NO Generation

The concentration of NO in the medium was measured using Griess reagent as an indicator of NO generation, as described previously [51]. RAW264.7 cells were split and seeded into a 96-well flat bottom microplate at a density of 2 × 10^4^ cells per well and incubated at 37 °C for 1 h. The cells were then treated with 1 µg/mL lipopolysaccharide (LPS) at various concentrations of LX519290. After 24 h incubation, NO concentration of the supernatants was measured by adding Griess reagent, thereafter, the absorbance of the mixtures was measured using a microplate reader (Victor3, PerkinElmer) at a wavelength of 520 nm.

### 4.7. Measurement of Intracellular ROS Generation

ROS production was evaluated using the ROS-responsive fluorescence indicator, 2′,7′-dichlorofluorescin diacetate (DCFH-DA), as described earlier [52]. To determine intracellular ROS scavenging activity, RAW 264.7 cells were split at a density of 2 × 10^4^ cells per well in a Lab-Tek chambered cover glass (Thermo Fisher Scientific, Waltham, MA, USA). After 24 h, the cells were treated with the samples of LX519290 and 200 μM *tert*-butyl hydroperoxide (*t*-BHP) and incubated for 2 h to induce ROS generation [53]. Subsequently, the cells were incubated with 20 μM DCFH-DA for 30 min and then analyzed under a confocal laser-scanning microscope (Carl Zeiss, Jena, Germany). Ascorbic acid was used as a positive control to compare with LX519290.

### 4.8. Activity of LX519290 Against Pro-Oxidant Challenge

To illustrate the antioxidant activity of LX519290 against pro-oxidant challenge, we measure H_2_O_2_ induced ROS generation using the ROS-sensitive fluorescence indicator DCFH-DA method with a slight modification [54]. In brief, RAW264.7 cells were first cultured in bottom transparent black 96-well plates (4 × 10^5^ cells /mL) for 24 h followed by treatment with varying concentrations of LX519290 and incubated for 30 min. Then, the medium was removed and the wells were mildly washed twice with PBS, and H_2_O_2_ (1 mM) was used as a pro-oxidant challenge for 30 min. Finally, DCFH-DA (40 µM) was added and incubated for 30 min at 37 °C in the dark. After incubation, DCF fluorescence intensity was analyzed at an excitation wavelength of 485 nm and an emission wavelength of 535 nm using a fluorometric plate reader (Victor3, PerkinElmer).

### 4.9. Total Thiol and Glutathione S-Transferase Measurement

Total thiol and glutathione *S*-transferases (GST) measurement was carried out according to the manufacturing protocol using a commercial kit (MAK151 for thiol quantitation, CS0410 for GST activity, Sigma-Aldrich, St. Louis, MO, USA).

### 4.10. AAPH-Induced Oxidative Stress in Rats

After one week of acclimatization, five random rats in each group were intraperitoneally administered AAPH (50 mg/kg) with antioxidants, ascorbic acid (150 mg/kg), or LX519290 (15 mg/kg) for 10 days. All drugs were dissolved in normal saline and prepared just before use. The no treatment (NT) group was injected with normal saline instead of AAPH and antioxidant compounds. After 10 days, blood samples were collected via heart puncture using a heparin-coated syringe, under carbon dioxide anesthesia for the ORAC assay [13] to determine the remaining antioxidant compounds in the plasma. Liver tissue was also collected and then analyzed by reverse transcription PCR (RT-PCR).

### 4.11. Reverse Transcription PCR Analysis of Gene Expression

Total RNA was isolated from RAW264.7 cells and liver tissues using TRIzol reagent (Life Technol., Gaithersburg, MD, USA), according to the manufacturer’s protocol. The RNA (1–10 μg) was transcribed into first-strand cDNA using an RT-&GO Mastermix (MP Biomedicals, Seoul, Korea), and the product was used as the PCR template. RT-PCR was performed using a Takara PCR thermal cycler and the following *SOD1*, *CAT*, *GPx1*, *NQO1*, and *GAPDH* (glyceraldehyde-3-phosphate dehydrogenase) primer sequences were used: *SOD1*: forward, 5′-AGG GCG TCA TTC ACT TCG AG-3′; reverse, 5′-TCC TTT CCA GCA GCC ACA TT-3′; *CAT*: forward, 5′-AGG CTC AGC TGA CAC AGT TC-3′; reverse, 5′-GCC ATT CAT GTG CCG ATG TC-3′; *GPx1*: forward, 5′-GCT CAC CCG CTC TTT ACC TT-3′; reverse, 5′-GAT GTC GAT GGT GCG AAA GC-3′; *GST*: forward, 5′-TGA GAG GAA CCA AGT GTT TGAG-3′ reverse, 5′- CAG GGG GAC TTT AGC TTT AGAA-3′; *HO-1*: forward, 5′-TGA GAG GAA CCA AGT GTT TGAG-3′; reverse, 5′-CAG GGG GAC TTT AGC TTT AGAA-3′; *NQO1*: forward, 5′-ATT GTA TTG GCC CAC GCA GA-3′; reverse, 5′-GCA CTC TCT CAA ACC AGC CT-3′; *Nrf-2*: forward, 5′-CTT TAG TCA GCG ACA GAA GGAC-3′; reverse, 5′-TCC AGA GAG CTA TTG AGG GACT-3′; *GAPDH*: forward, 5′-GCG AGA TCC CGC TAA CAT CA-3′; reverse, 5′-AGT GAT GGC ATG GAC TGT GG-3′. Genes for *SOD1*, *CAT*, *GPx1*, *NQO1*, and *GAPDH* were amplified with a denaturation step at 94 °C for 30 s, an annealing step at 58 °C for 30 s, and an extension step at 72 °C for 30 s for 30 cycles. The mRNA levels were normalized to the housekeeping gene, *GAPDH*.

### 4.12. Statistics

All experiments were basically carried out in triplicate and the results are expressed as the mean ± standard deviation (SD). Statistical significance was determined by a Student’s *t*-test or one-way analysis of variance (ANOVA), using the program IBM SPSS statistics. When the data from the ANOVA were significant, the differences in antioxidant activity among the LX519290 samples were analyzed by a post hoc test, either Tukey’s or Duncan’s test [7]. The critical level for statistically significant results was defined as *p* < 0.001, *p* < 0.05, or *p* < 0.01.
